# Optimizing clinical and organizational practice in cancer survivor transitions between specialized oncology and primary care teams: a realist evaluation of multiple case studies

**DOI:** 10.1186/s12913-017-2785-z

**Published:** 2017-12-16

**Authors:** Dominique Tremblay, Catherine Prady, Karine Bilodeau, Nassera Touati, Maud-Christine Chouinard, Martin Fortin, Isabelle Gaboury, Jean Rodrigue, Marie-France L’Italien

**Affiliations:** 1 Centre de recherche - Hôpital Charles-Le Moyne, Centre intégré de santé et de services sociaux de la Montérégie-Centre, 150 Place Charles-Le Moyne, Longueuil, Québec, (J4K 0A8) Canada; 2 Campus de Longueuil - Université de Sherbrooke - Faculté de médecine et des sciences de la santé, 150 Place Charles-Le Moyne, Longueuil, Québec, (J4K 0A8) Canada; 3 Centre intégré de santé et de services sociaux de la Montérégie-Centre, 3120 Boulevard Taschereau, Greenfield Park, Québec, (J4V 2H1) Canada; 4 Université de Montréal – Faculté des sciences infirmières et Centre d’innovation en formation infirmière, 2375 Chemin Côte-Ste-Catherine, Montréal, Québec, (H3T 1A8) Canada; 5 École Nationale d’Administration Publique, 4750 Avenue Henri-Julien, 5è étage, Montréal, Québec, (H2T 3E5) Canada; 6 Université du Québec à Chicoutimi – Département des sciences de la santé, 555 Boulevard de l’Université, Chicoutimi, Québec, (G7H 2B1) Canada; 7 Centre intégré universitaire de santé et de services sociaux du Saguenay-Lac-Saint-Jean, Hôpital de Chicoutimi, 305 St-Vallier, Chicoutimi, Québec, (G7H 5H6) Canada; 8 Université de Sherbrooke - Département de médecine de famille et de médecine d’urgence, 3001 12e Avenue Nord, Sherbrooke, Québec, (J1H 5N4) Canada

**Keywords:** Case study, Coordination, Intervention, Mixed methods, Primary care, Risk-based cancer care, Realist evaluation

## Abstract

**Background:**

Cancer is now viewed as a chronic disease, presenting challenges to follow-up and survivorship care. Models to shift from haphazard, suboptimal and fragmented episodes of care to an integrated cancer care continuum must be developed, tested and implemented. Numerous studies demonstrate improved care when follow-up is assured by both oncology and primary care providers rather than either group alone. However, there is little data on the roles assumed by specialized oncology teams and primary care providers and the extent to which they work together. This study aims to develop, pilot test and measure outcomes of an innovative risk-based coordinated cancer care model for patients transitioning from specialized oncology teams to primary care providers.

**Methods/design:**

This multiple case study using a sequential mixed-methods design rests on a theory-driven realist evaluation approach to understand how transitions might be improved. The cases are two health regions in Quebec, Canada, defined by their geographic territory. Each case includes a Cancer Centre and three Family Medicine Groups selected based on differences in their determining characteristics. Qualitative data will be collected from document review (scientific journal, grey literature, local documentation), semi-directed interviews with key informants, and observation of care coordination practices. Qualitative data will be supplemented with a survey to measure the outcome of the coordinated model among providers (scope of practice, collaboration, relational coordination, leadership) and patients diagnosed with breast, colorectal or prostate cancer (access to care, patient-centredness, communication, self-care, survivorship profile, quality of life). Results from descriptive and regression analyses will be triangulated with thematic analysis of qualitative data. Qualitative, quantitative, and mixed methods data will be interpreted within and across cases in order to identify context-mechanism associations that explain outcomes.

**Discussion:**

The study will provide empirical data on a risk-based coordinated model of cancer care to guide actions at different levels in the health system. This in-depth multiple case study using a realist approach considers both the need for context-specific intervention research and the imperative to address research gaps regarding coordinated models of cancer care.

**Electronic supplementary material:**

The online version of this article (10.1186/s12913-017-2785-z) contains supplementary material, which is available to authorized users.

## Background

The anticipated 79% increase in cancer survivors over the next two decades, coupled with the scarcity of human and financial resources [1] threatens to produce a crisis in health systems across Canada and in most industrialised countries [2, 3]. One way to potentially avert this crisis is by implementing and evaluating integrated care models that, while taking cancer-related risks into account, enable more fluid transitions between specialized cancer care teams and primary care providers (PCPs) [4–6]. Currently, the siloed functioning of health systems hinders these transitions [7, 8], leaving them incomplete, highly variable and subject to informal agreements [9]. Badly coordinated transitions are at root of unmet needs that have negative consequences on health and quality of life [10]. As a result, survivors struggle with the human and economic burden of living with cancer as a chronic disease [11, 12], a burden it is now essential to alleviate.

The incidence of cancer and other chronic diseases increases with age. An increasing number of adults and seniors are living for many years with cancer alongside other chronic diseases (e.g. diabetes, heart disease, osteoporosis, depression). Care models must be better adapted to the realities of a triple burden that combines cancer survivorship, chronic disease [11, 13] and multimorbidity [10, 14]. All cancer survivors require follow-up to manage the effects of cancer and its treatment, screen for recurrence or the appearance of a new cancer, and coordinate care [15]. About half of all cancer survivors should, at minimum, undergo regular assessment of their global state of health (information, symptom management), while a third will require additional support (peer support, health education). Between 35% and 40% of survivors will need expert support to manage symptoms and distress, while 10% to 15% will require close follow-up and more complex interventions [16]. This follow-up care must consider the physical, psychological and side effect aspects of survivorship [16], as well as multimorbidity [8, 17]. This is especially important during transitions between specialized cancer care and primary care with a family physician [8, 9, 15]. Coordinated care models are therefore sought by both clinicians and survivors to ensure that needs are met in the right way, at the right time, by the most appropriate professional [6].

The American Society of Clinical Oncology (ASCO), the Canadian Cancer Research Alliance (CCRA), the Canadian Partnership Against Cancer (CPAC) as well as a vast literature review suggest that a model of integrated care involving cancer care teams and primary care providers (PCPs), and adapted to a person’s cancer-related risk, could make the most of the expertise of each group of professionals [4–6, 9, 18]. Such a model requires communication through a survivorship care plan between members of the cancer care team and PCPs [19]. ASCO also highlighted the importance of supporting “demonstration programs to test models of coordinated, interdisciplinary survivorship care in diverse communities and across systems of care” (p.638) [4]. Oncological risk (low, moderate or high) is based on several factors, including the types of treatment received, their toxicity, and the risk of recurrence [20]; an assessment of risk contributes to determining the appropriate level of care, criteria for medical and psychosocial referrals, and the resource(s) most able to meet a person’s needs [20].

Despite these recommendations, the roles, responsibilities and scope of practice of the various professionals participating in survivorship care remain ill-defined [6, 10, 21]. Professionals in specialized teams do not always feel equipped to respond to the totality of survivor needs, nor, in overcrowded ambulatory clinics, do they always have the time [10]. For their part, PCPs, notably family physicians, consider they lack training in the follow-up of cancers, which involve more than 200 different diagnoses and increasingly complex treatments [6, 22]. Furthermore, Grunfeld’s work demonstrates that there is no added value in having survivorship care provided by cancer specialists rather than PCPs [23–27]. Given the haphazard, suboptimal and fragmented care currently available, and the recommendations from authorities in oncology, the present project will produce robust evidence, applicable for rapid use in different contexts, on a coordinated care model that takes into account survivors’ cancer-related risk and potential multimorbidity.

### Study aim and research questions

The goal is to develop, analyze and implement a demonstration project and evaluate the outcomes of a Risk-based Coordinated Cancer Care Model (referred to hereafter by the acronym RbCCCM) that focuses on cancer survivors’ transitions between specialized oncology teams and primary care teams. This goal is formulated according to the Medical Research Council’s stages of development for complex interventions [28–30], and is specifically adapted to people with chronic diseases [31]. The study will address the following questions:

1. What are the contextual factors and mechanisms that most effectively support a RbCCCM?
2. How, by whom, for whom and under what conditions is the model translated into clinical and organizational practice?
3. What are the model’s effects on professionals and patients?
4. In what ways do context and mechanisms create conditions that are favourable (or not) to producing the outcomes of the RbCCCM?

Answering these questions involves mobilizing the components of our theoretical framework and taking a participatory approach, involving researchers/front-line actors/patients, inspired by intervention research [32–34]. Our working hypothesis is that a RbCCCM involving specialized cancer care teams and primary care teams will have a positive effect on professional practice and, in the end, on the survivors’ experience of transitions between teams.

### The intervention

The RbCCCM is a novel intervention with multiple components: professional, organizational, client-based (patients and families), governance (Table 1). To address the fragmentation of care, the RbCCCM relies on activities that promote coordinated clinical and organizational practices and on the behaviour of survivors during the transition from oncology care to primary care [35, 36]. The RbCCCM incorporates consideration of cancer-related risks that may combine with multimorbidity in certain patients [31].

**Table 1:** Components of the RbCCCM^a^

Components	Planned activities	Deliverables
Professional	• Identification of needs (e.g..: continuing education, clinical tools^b^, skills development) • Oncological risk evaluation and multimorbidity (health status) • Clarification of roles	• Online training modules • Criteria for referrals between teams • Identify and develop clinical tools (survivorship care plan)
Organizational	• Development/adaptation of coordination and communication tools • Consolidation of intra- and inter-team work	• Identify organizational actions for teamwork • Design effective service corridors
Clientele^c^	• Development/adaptation of information tools • Identification of community resources • Development/adaptation of education for clientele (self-care, symptom management, navigation in health system)	• Paper and web publications • Survivorship workshop • Adapted monitoring tools • Inventory of resource persons
Governance (policy makers, administrators)	• Establishment of normative and legislative aspects related to survival issues • Establishment/consolidation of a provincial survivorship committee (mandates and responsibilities)	• Recommendations/incentives • Recognition/clinical time for survivorship care • Mention of RbCCCM in provincial and national cancer and action plans

^a^To be validated in Step I: development of the intervention with the decision–making partners

^b^Ongoing procedures for translating clinical reference tools from the *Oncology Nursing Society*

^c^Clientele refers to cancer patients and their families

The intervention involves four interrelated steps: I) development of a RbCCCM based on evidence around survivorship care models and an environmental scan of the Québec context; II) implementation of a demonstration project; III) measurement of the model’s effects on professionals and survivors; and IV) evaluation of conditions required for the RbCCCM to produce these effects.

### Analytical framework of the RbCCCM

**Fig. 1:**
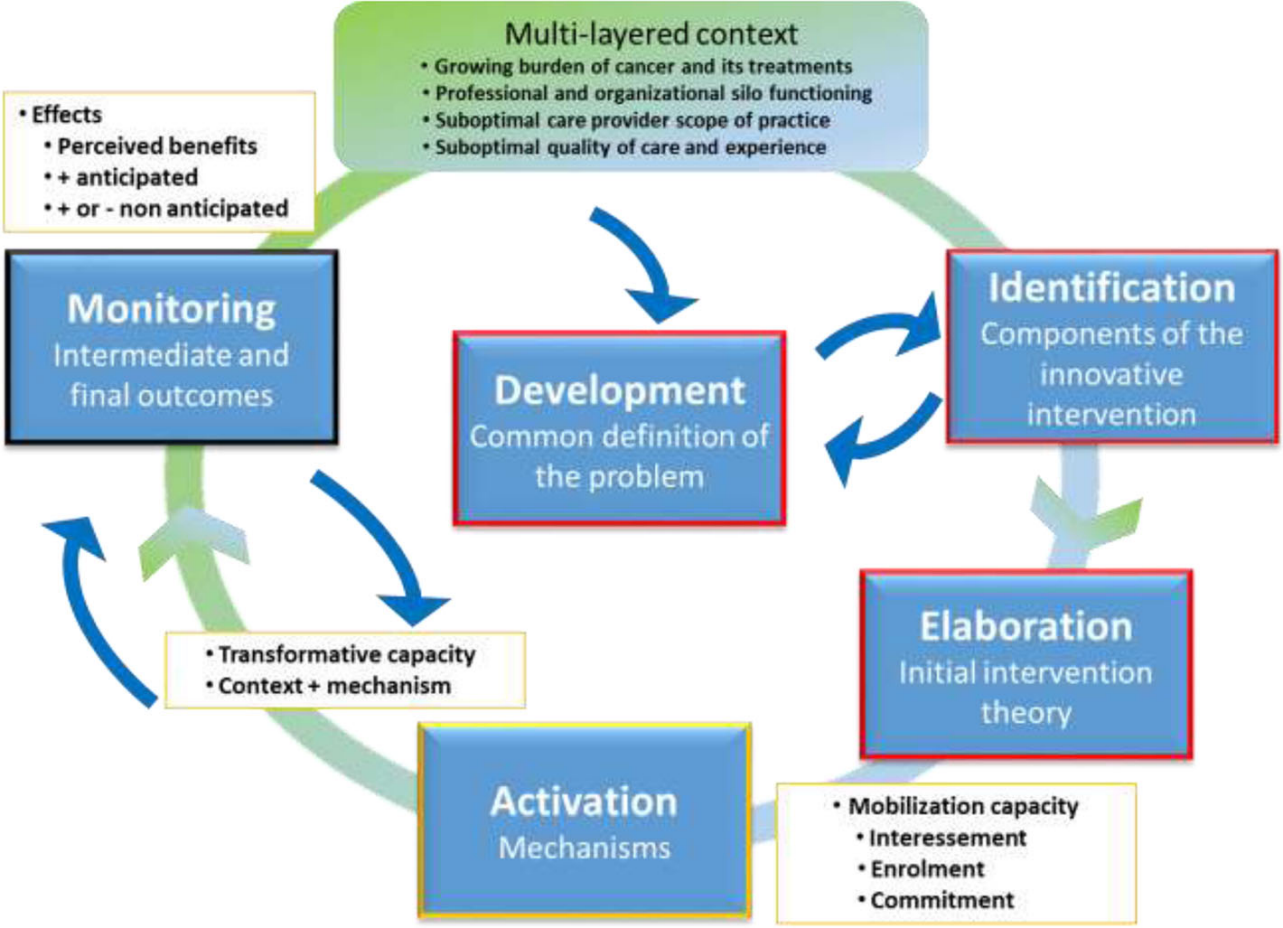
Framework developed for the analysis of the RbCCCM intervention

The analytical framework (Fig. 1) combines theoretical work and empirical evidence from Tremblay et al.’s previous research [37, 38]. It is inspired by aspects of actor-network theory (ANT), which, very briefly, holds that translating a novel intervention into practice depends on the ability of multiple actors — with diverse interests, needs and objectives that are often in competition — to work together towards a common goal [39–41]. The construction of this network to support an intervention (the RbCCCM in our case) proceeds in four key stages along the path from idea to practice: contextualization (identification of actors/resources and the play of influence at different levels); problem definition (discussion/decision-making processes to clarify the problem and its solution, determine mechanisms that need to be activated and the initial intervention theory); mobilization, whereby actors become interested in the innovation (interessement), assume roles (enrolment) and commit (commitment) to achieving the anticipated outcomes of the innovation and monitoring processes and effects [42]. To render the ANT more concrete in operational terms, the framework includes principles of realist evaluation [43–46]. Realist evaluation is an approach that brings together: 1) the multi-level context and its influence on professional and organizational practice as well as the experience of people living with cancer (contextualization (C)) [47]; 2) development of a common definition of the problem and identification of the novel intervention’s components, leading to the development of an initial theory of RbCCCM (problem definition); 3) activation of mechanisms (M) in terms of people’s interessement, enrolment and commitment to the RbCCCM (mobilisation); and 4) monitoring of anticipated or unanticipated outcomes (O) in professional and organizational practice and the experience of cancer survivors (monitoring). The successful translation of the intervention (RbCCCM) into practice would involve the achievement of positive effects without producing unanticipated negative effects. The arrows in the figure illustrate that the translation process is not necessarily linear. For more details see Additional file 1.

## Methods/design

**Fig. 2:**
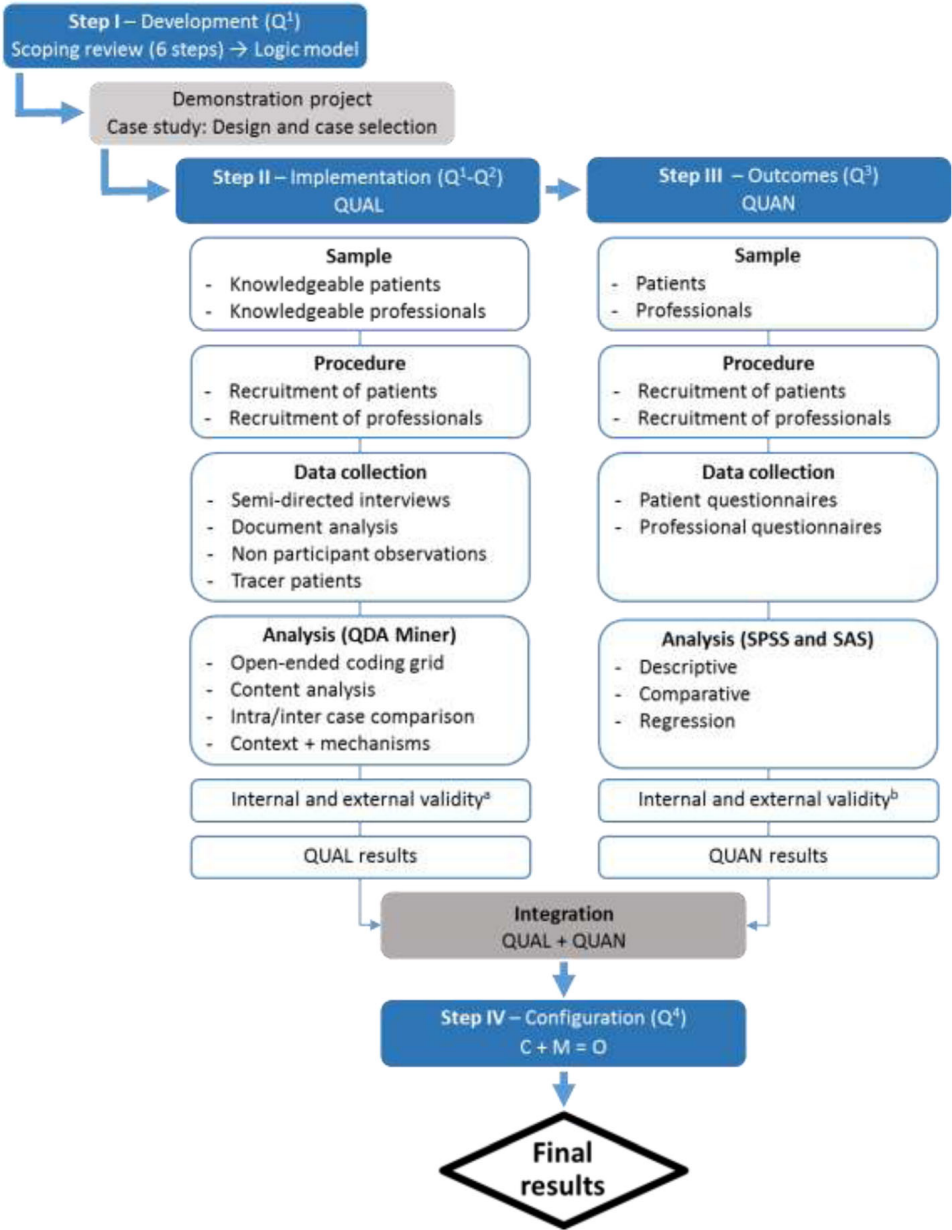
Graphic illustration of the methodological approach. Legend: Q1-Q4: Research questions; QUAL and QUAN refer respectively to qualitative and quantitative data; a Refers to the definitions of Miles MB, Huberman M, Saldana J. Qualitative data analysis. A Methods Sourcebook. 3 ed. Thousand Oaks: Sage, 2014, p. 278-279; b Refers to the definitions of Creswell JW. Research Design: Qualitative, Quantitative, and Mixed Methods Approaches. 3 ed. Thousand Oaks: SAGE Publications; 2009, p.162; C: Context, M: Mechanisms, O: Outcomes

The methodological approach (Fig. 2) is adapted to our research questions and to the stages involved in development, implementation, and evaluation of the intervention [48], along with conditions required to produce the outcomes of the RbCCCM. Methods have also been strategically chosen for pragmatic qualities suited to interventional research (Table 2). The design involves a multiple case study based on qualitative data that draws on the principles of realist evaluation and quantitative data in an experimental approach.

**Table 2:** Elements involved in steps I to IV and data collection

	Professionals				
Elements	Policy makers, administrators	PCP	Onco.	Patients, families	Documents
Step I - Development					
• Scientific evidence: scoping study	PWG	PWG	PWG	PWG	SDB
• Environmental scan: identification of actors, issues, needs	PWG	PWG	PWG	PWG	
• Development of the initial theory of the intervention	PWG	PWG	PWG	PWG	
• Production of the logic model of the intervention	PWG	PWG	PWG	PWG	
Step II - Implementation					
• Adoption/adaptation into practice	II	II, O	II, O	TC	LR
• Description of ongoing incentives, mobilization, commitment	II	II	II	TC	LR
• Identification/description of mechanisms for translation into practice	II	II, O	II, O	TC	LR
• Identification/description of facilitating and constraining factors/reduction of barriers	II	II, O	II, O	TC	LR
Step III – Outcomes					
*For professionals*					
• Scope of practice; teamwork; work environment		S	S		
• Shared leadership; density/centrality of the cancer network		S	S		
• Socio-demographic characteristics		S	S		
*For patients*					
• Responsiveness; self-care capacity; quality of life; out-of-pocket costs				S, PD	
• Socio-demographic and clinical characteristics				S	
Step IV- Configuration					
*Global evaluation*					
• Context-Mechanisms-Outcomes (C + M = O)	IVI	IVI	IVI	IVI	

*Legend*: *LR* Literature review, *II* Individual interview, *IVI* Individual validation interview, *PWG* Participative working group, *O* Non-participant observation, *S* Survey, *TC* Tracer cases, *SDB* Scientific data bank, *PD* Patient diary

### The RbCCCM intervention

#### Step I - Development

Development of the RbCCCM begins by gathering evidence [30, 48] on integrated survivorship models (components, activities, professionals involved, nature and scale of effects, theoretical bases) [49] and the concept of cancer-related risk. This will be followed by an environmental scan of existing local initiatives in Québec to promote coordination between cancer care and primary care. This step will provide answers to Question 1.

A scoping review will be undertaken in six stages [50, 51]: 1) identify the research question; 2) identify relevant studies; 3) select studies according to inclusion and exclusion criteria; 4) classify evidence by theme and challenge area; 5) assemble, summarize and report results; and 6) consult potential users to determine where we are with transitions (contextualisation), where we want to be (problem definition) and what is needed to get there (mobilization) and what outcomes are anticipated (monitoring).

These steps are in line with the analytical framework presented in Fig. 1. We will use the Nose to Tail Tool (NTT) internet platform [52], which enables deliberative processes (participatory group work) around the development and planning of innovative interventions, the context in which they will be used, and the decision to move ahead with implementation. This will involve key informants from the primary care and nursing committee of the *Direction générale de cancérologie du Québec*, managers and clinicians from cancer care teams, and Family Medicine Groups (FMGs).

The deliverable is the concerted production of the RbCCCM logic model [53] that will specify contextual factors and challenges, training needs of professionals, and resources available (or possible to recruit) to implement the RbCCCM. Finally, depending on results, accredited online training modules (cancer and treatments, interdisciplinary care, survivorship, assessment of cancer-related risk, multimorbidity, health promotion, professional and patient resources) will be developed or adapted. An expert recognized for clinical leadership will be recruited to adapt or develop training to support clinical judgement in survivorship interventions and provide coaching. The training will be offered in collaboration with the leader of the nursing committee at the *Direction générale de cancérologie*.

### Demonstration project

The design for analyzing the implementation (Step II) and evaluation of outcomes (Step III) is a multiple case study [54] based on qualitative and quantitative data [55]. The design is appropriate to understanding a contemporary phenomenon (the RbCCCM) as it manifests in the real world where it may be influenced by multiple changing and interdependent factors and contextual challenges. A case is defined as the cancer care trajectory in order to understand how the RbCCCM mobilizes members of specialized cancer care teams and primary care teams during transitions.

The selection of cases is based on theory [54] to represent real-world differences [56] in terms of geographic location (the territorial expanse served; rural/semi-urban/urban areas), mission (academic or not), the size and diversity of care teams and their interest in being involved in the RbCCCM demonstration project. Units of analysis are the hospital-based cancer care teams and the FMGs of two Integrated Health and Social Service Centres (3/11 FMGs + one hospital in Saguenay-Lac-Saint-Jean (Case 1); 3/31 FMGs + one hospital in the Montérégie (Case 2). The selection of cases is also influenced by feasibility and our knowledge of these establishments gained through their participation in prior research we conducted on cancer care [8, 37, 38, 57–63], on primary care [64, 65], as well as on organizational factors that influence the care experience of people having received a cancer diagnosis [38].

Case 1 is an academic health centre that has deployed, since 2010, an intervention in FMGs aimed at improving care coordination for people with chronic diseases (other than cancer) through nurse case managers and group sessions to support self-management [64, 65]. In this case, the RbCCCM might be integrated into these existing practices, with cancer treated as a chronic disease [13]. Case 2 has, since 2001, deployed efforts in specialized cancer care (nurse navigators, implementation of a cancer care network, development of an integrated centre, introduction of the clinician advisor role) [37, 38, 60, 63, 66, 67]. The two cases offer possibilities for analyzing the RbCCCM and better understanding variations in implementation and outcomes. The realist approach is employed for its pragmatic and participatory principles that help to discover what works (and what doesn’t), how it works, for whom, and under what conditions. This approach is suited to analysis of complex interventions [48].

#### Step II – Implementation (pilot test)

Analysis of the implementation of the RbCCCM [30] will enable us to explain how and why the program is translated into clinical and organizational practices, with whom, for whom, and under what conditions. It will focus on the four moments of translation of the ANT to see how actors relate to the RbCCCM. In concrete terms, the analysis will reveal the “pros” and “cons” with regard to the RbCCCM and how these controversies are resolved to recognize the RbCCCM as a necessary bridge towards coordinating survivorship care during transitions. This step will explain how cooperative mechanisms are activated by the development of mutual trust and sharing of expertise, leadership, and the use of coordination and communication tools (e.g. professional time, referral criteria, clinical practice guidelines, problem detection tools) in a given context. This step will complete the response to Question 1 and will answer Question 2.

#### Sampling (step II)

Participants are people living with cancer, and members of the cancer care teams and FMGs involved in treating these patients.

The patient sample will be made up by survivors who have been diagnosed with cancer and have completed active treatment. Inclusion criteria are: to have been diagnosed with cancer (breast, colorectal, prostate), to have completed active treatment, to have received over the past 12 months or currently be receiving care other than palliative care in one of the outpatient cancer clinics involved in the study (Montérégie or Saguenay-Lac-Saint-Jean), to have a family physician belonging to one of the participating FMGs, and to be able to read and speak French. The types of cancer have been selected based on age-related incidence and 5-year survival rates (88% for breast, 64% for colorectal, and 96% for prostate) [1].

The professional sample will be made up by members of cancer care and FMG teams (family physicians, specialist physicians, nurses, other professionals and managers). Inclusion criteria are: to be a family physician working in a FMG where the client base includes people living with cancer, a specialist physician affiliated with the oncology department of a participating site, a nurse (nurse navigator, clinical nurse specialist or nurse practitioner in a FMG) or another professional working with people living with cancer.

#### Procedure (step II)

Project collaborators and partners are committed to facilitating the recruitment of professionals and patients. For patients, a locally-designated person will identify potential participants and provide them information about the study. Interested patients will be invited to sign an “Authorization to be contacted” and a member of the research team will provide them the information required for informed consent. For professionals, an email message inviting them to participate will be sent out via internal email lists, and will include a link to a brief video (3 min) presenting the study (objectives, duration of the interview, type of questions asked). A positive response to the email will be followed by setting an appointment for the interview at the participant’s convenience. Compensation to a maximum of $100 is anticipated for the time involved in the interview (professionals and patients). The amount will be adjusted to the duration of the interview when participants indicate availability of less than one hour.

#### Qualitative data collection (step II)

Qualitative data will be collected from a number of sources: semi-directed interviews with key informants (patients, professionals, managers) (*n* = 12 per case) [68], document analysis (tools used in care coordination and continuity, chart review, meeting minutes) [69] and observation (follow-up appointments, team meetings). Tracer patients, referring to patients followed throughout the healthcare process [70, 71], will enable documentation of transitions in real time (interviews, observation, clinical chart). The selection of tracer patients (*n* = 16) will be based on cancer type (breast, colorectal, prostate) and affiliation with a family physician in a FMG involved in one the two cases of the study. Patients over 70 years of age will make up 50% of tracer cases given their increased potential for multimorbidity [72], the recognition of unmet needs in this group [73] and the challenges of an integrated oncogeriatric approach [67]. Professionals involved in the care of these tracer patients will be interviewed to incorporate views on the context and mechanisms (active ingredients) of real-world care transitions from people directly involved in the RbCCCM.

#### Qualitative data analysis (step II)

Systematic and iterative content analysis will involve listening to the audio of interviews, reading and coding all data [54] integrated into a QDA Miner database. A semi-open analytical grid, based on the analytical framework (Fig. 1) will enable an initial coding structure and the addition of new codes during analysis [74]. Each case will be analyzed separately, followed by an inter-case analysis to highlight recurring models, differences and similarities between the cases (semi-regularities) [43].

#### Validity (step II)

Validity is increased through the triangulation of multiple data sources [54] and the interactions between researchers and potential knowledge users. Collaborative coding based on solid theory and concepts, along with validation of findings by users will contribute to internal validity (credibility). The detailed description of the RbCCCM and cases will contribute to external validity (transferability) and the pragmatic aims of the study.

#### Step III - Outcomes of the RbCCCM

This stage aims to understand the outcomes of the RbCCCM in patients and professionals in each of our cases. It uses experimental approaches to verify our working hypothesis that the RbCCCM will have a positive impact on professional practice and the care experience of cancer survivors (patient-reported experience). The impossibility, for ethical and professional reasons, of conducting a randomized controlled trial calls for a pragmatic before-and-after design with delayed intervention (6-month follow-up) where teams in one case will provide usual care (control segment) while teams in the other case are implementing the components of the intervention (intervention segment). This step will answer Question 3.

#### Sample (step III)

The sample size is calculated according to our previous studies on the responsiveness of cancer care [75] and the Health Education Impact Questionnaire (heiQ) scoring for self-management capacity [76]. Given that scores on the scales range between 1 and 4, an average score of 2.3 and a difference of 0.3 are reasonable. One hundred patients per case will be recruited, based on anticipated attrition of about 10%. If we consider the heiQ distress score, this sample size would enable us to detect a 0.3 difference between measures at 2 time points in a same group, and between groups, with a standard deviation of 0.71 and confidence interval of 0.80.

The sample size will include about 50 professionals per case (100 total) including personnel in the FMGs and the cancer care teams. The minimum response rate based on our previous studies is anticipated at 40%. Limitations related to the small sample size will, as is common in case studies, be compensated by the qualitative data described in Step II [77].

#### Procedure (step III)

The patient recruitment process is based on our previous studies. It relies on collaboration from care teams to help us identify potential patient participants. Standardised criteria and recruitment procedures will be used and a local professional (the research nurse in our study) will be designated to explain the study and obtain informed consent. If the patient agrees to participate, they will be given a postage-paid envelope, information letter and printed questionnaire. Patients may also choose to complete the questionnaire on line (SurveyMonkey Inc., San Mateo, California, USA). In this case, patient participants will be given an access code. Questionnaires are anonymous and managed to respect confidentiality. Reminders and a compensation of $25 aim to increase response rates.

All professionals working in specialized cancer care and primary care teams will receive an invitation by email that includes a link to a short video explaining the study objectives and expectations of participants. The questionnaire will be provided in print and on line (SurveyMonkey Inc., San Mateo, California, USA). Three reminders at two-week intervals, along with marketing through communities of practice, scientific networks (e.g. RRISIQ, Réseau 1, CANO) and social media, along with compensation of $25, aim to increase response rates [78].

#### Quantitative data collection (step III)

Anticipated outcomes in patients will be measured before the intervention (T0) and six months after the start of the RbCCCM (T1). A self-administered questionnaire (30 min) will measure the patient’s perception of their experience of care during transitions between teams: responsiveness [75]: timeliness of access (4 items; α = 0.77), person-centred care (5 items, α = 0.67), quality of communication (5 items, α = 0.85), self-management capacity based on the heiQ validated in oncology [76], involving: emotional distress (6 items; α = 0.77), wayfinding in the health system (5 items, α = 0.85), social support and integration (5 items, α = 0.85), as well as quality of life SF12 (12 items, α = 0.83) [79]. Though the project does not include an economic evaluation, data related to some costs will be collected, notably among men with prostate cancer [80], using a generic instrument and a diary to report costs of using health care (time, transport costs, non-reimbursed medication costs, lost income). This data will provide the basis for economic analysis in a future study. Sociodemographic and clinical data will be collected: age, gender, comorbidity, cancer type, health status, chronic diseases [81], survivorship profile [82].

Anticipated outcomes among professionals will be measures before the intervention (T0) and 6 months after the start of the RbCCCM (T1), an interval based on feasibility in a demonstration project. A self-administered questionnaire (30 min) with validated psychometric properties will be employed: SCOP scope of practice (26 items, α = 0.89) [83], teamwork (5 items, α = 0.84) [84], team environment (19 items, α = 0.88–0.93) [85], shared leadership (13 items, α = 0.66) [86]. The density and centrality of intra- and inter-team links will be measures using social network analysis [87]. Data on sociodemographic characteristics will also be collected (age, gender, profession, practice setting, years of experience).

#### Quantitative data analysis (step III)

Descriptive statistical analyses, comparative analyses based on data type (t-test, Chi2, ANOVA, ANCOVA), and regression analyses (multilevel logistic and linear because of the structure and hierarchy of data) will be conducted. These will enable us to determine the association (positive, negative or neutral) between the RbCCCM, professional practice, and the care experience of cancer survivors during transitions, while controlling for certain potentially confounding individual and organizational variables. The threshold for significance will be 5%.

#### Validity (step III)

Internal validity rests on use of questionnaires with validated psychometric strengths, sample size, and control of confounding variables in the regression models. Results will be generalizable to other settings with characteristics similar to the cases in the study.

#### Step IV – Analysis of conditions required for the production of outcomes

The outcomes of the RbCCCM depend on a number of contextual, human and clinical factors that may act synergistically or antagonistically. A configurational approach is suited to the analysis of conditions that underlie production of the outcomes in complex interventions [88] in order to understand critical factors in the RbCCCM that contribute to developing lasting fluidity in the transitions between care teams. Realist evaluation provides the methodological basis, stipulating that the association between context (C) and mechanisms (M) produces the outcomes of an intervention (O): C + M = O [43]. These configurations represent semi-regularities: “regularities” because they are recursive models with strong explanatory potential; “semi” because these recursive models are highly dependent on context and can vary. This step will answer Question 4.

Results from qualitative analysis (C + M drawn from Steps I and II) and quantitative analysis (outcomes measured in Step III) will be combined [89] in order to develop the C + M = O associations of the RbCCCM [43, 88]. All data will be integrated into a database in QDA Miner (Provalis Research) to produce configurations that offer a clear, rich and detailed understanding of the RbCCCM as an innovative intervention, providing a refined theory of the intervention. Results will be validated pragmatically among researchers, front-line actors and patient participants. The validity of data integration rests on quality criteria from the Mixed Method Appraisal Tool available on WIKI [90] and on perceptions of actors in the field.

### Knowledge transfer plan

In line with the Knowledge to Practice framework [91], our knowledge exchange strategy considers the particular needs of each knowledge user. The research approach therefore involves key informants and users in the development of the RbCCCM intervention. This strategy enables the targeting of significant evidence to potential users and the adaptation of the intervention to local contexts (clinical tools, referral criteria). As well, the inclusion of knowledge users and partnership with a cancer survivor on the research team enable the identification of facilitators and impediments (e.g. lack of knowledge about survivorship care) to the implementation of the RbCCCM. Effective strategies to overcome these obstacles may then be used as educational interventions (accredited continuing medical education) or as interventions led by patients (e.g. patient awareness of the RbCCCM leads them to request a survivorship plan from their specialist). To promote and deepen exchanges, knowledge users will be informed on a regular basis about progress on the project (e.g. newsletter, agenda item on monthly clinical meetings). As well, the research team will organise (1×/year) a half-day knowledge exchange symposium on themes related to the RbCCCM (presentation of scoping review, preliminary results).

Components of the Knowledge to Practice framework are the cornerstone of our integrated knowledge transfer (IKT) plan [91] and are suitable for the study of innovative interventions. This framework defines knowledge uptake as a dynamic and recursive process that involves the synthesis, dissemination, exchange and uptake of knowledge with a view to improving care and strengthening the health system. This process takes place within a network of complex interactions between researchers and knowledge users that vary in intensity, complexity and commitment depending on the nature of the research and its results, as well as the particular needs of each knowledge user. It is therefore consistent with interventional research. The framework has two central components. The first draws on the notion of the funnel, where the creation of knowledge involves its progressive refinement to facilitate adoption by actors in the field. The second represents the active part of the process as a cycle leading to the implementation or application of research findings. This second component involves seven stages that may be sequential or occur in feedback loops: 1) problem identification, 2) adaptation of knowledge to local context, 3) assessment of barriers to knowledge use in that context, 4) selection, adaptation and implementation of interventions, 5) monitoring of knowledge use, 6) evaluation of results, and 7) maintenance of knowledge use. The framework is perfectly aligned with the analytical framework developed in this project (Fig. 1) and will guide us towards the interventional research goals.

## Discussion

### Challenges and mitigation strategies

Potential difficulties in this project are: 1) the engagement of professionals and their willingness to collaborate; 2) recruitment of patients; 3) the distance separating the two cases; 4) the large scope of the project. The following strategies will be adopted to reduce attendant risks: first, we have secured the participation of research nurses, remunerated by the project and recognized in each of the case settings for their clinical leadership and coaching abilities [92]. They will be able to quickly identify problems that arise with the implementation of the RbCCCM, work with the research team to find solutions suitable to the local context, and counsel team members on complex cases. Second, institutional leadership support for the project creates winning conditions. Québec institutions must periodically design and evaluate their cancer action plans, and care and service continuity is a priority in these plans. Finally, the presence of the RbCCCM will be seen by teams as a useful and supportive project.

The study will enable us to develop a Risk-based coordinated cancer care model (RbCCCM), to analyze the implementation of a demonstration project, and to evaluate the outcomes of the RbCCCM. It will allow us to test the RbCCCM in various practice communities across the health system. Anticipated results will: 1) identify factors that facilitate or impede a RbCCCM, 2) evaluate how the model is translated into clinical and organizational practice, 3) evaluate outcomes in professionals and patients, and 4) identify contexts and mechanisms that create favourable and unfavourable conditions for the production of RbCCCM outcomes. This will provide essential information for decision-makers and managers about the roles and responsibilities assumed by cancer and primary care teams in the provision of care and at transition points along the cancer care continuum. In addition, the project will increase awareness among a broad range of stakeholders (policy-makers, managers, professionals, services users) of factors that help to activate promising mechanisms involved in a coordinated model of cancer care.

## Additional file

Additional file 1: Framework developed for the analysis of the RbCCCM intervention and definitions. (DOCX 280 kb)

## Abbreviations

ANT: Actor-network theory
ASCO: American Society of Clinical Oncology
CANO: Canadian Association of Nurses in Oncology
CCRA: Canadian Cancer Research Alliance
CPAC: Canadian Partnership Against Cancer
FMG: Family Medicine Group
IKT: Integrated knowledge transfer
NTT: Nose to Tail Tool
PCP: Primary care providers
RbCCCM: Risk-based Coordinated Cancer Care Model
RRISIQ: Réseau de recherche en interventions en sciences infirmières du Québec/Quebec Network on Nursing Intervention Research
