# Supplementary material for: Optimizing clinical and organizational practice in cancer survivor transitions between specialized oncology and primary care teams: a realist evaluation of multiple case studies
Source: BMC Health Serv Res. 2017 Dec 16;17:834. doi: 10.1186/s12913-017-2785-z (PMC5732430; doi:10.1186/s12913-017-2785-z)
Supplement: Additional file 1: — Framework developed for the analysis of the RbCCCM intervention and definitions. (DOCX 280 kb) [file 12913_2017_2785_MOESM1_ESM.docx]

**Additional file 1** Framework developed for the analysis of the RbCCCM intervention and definitions


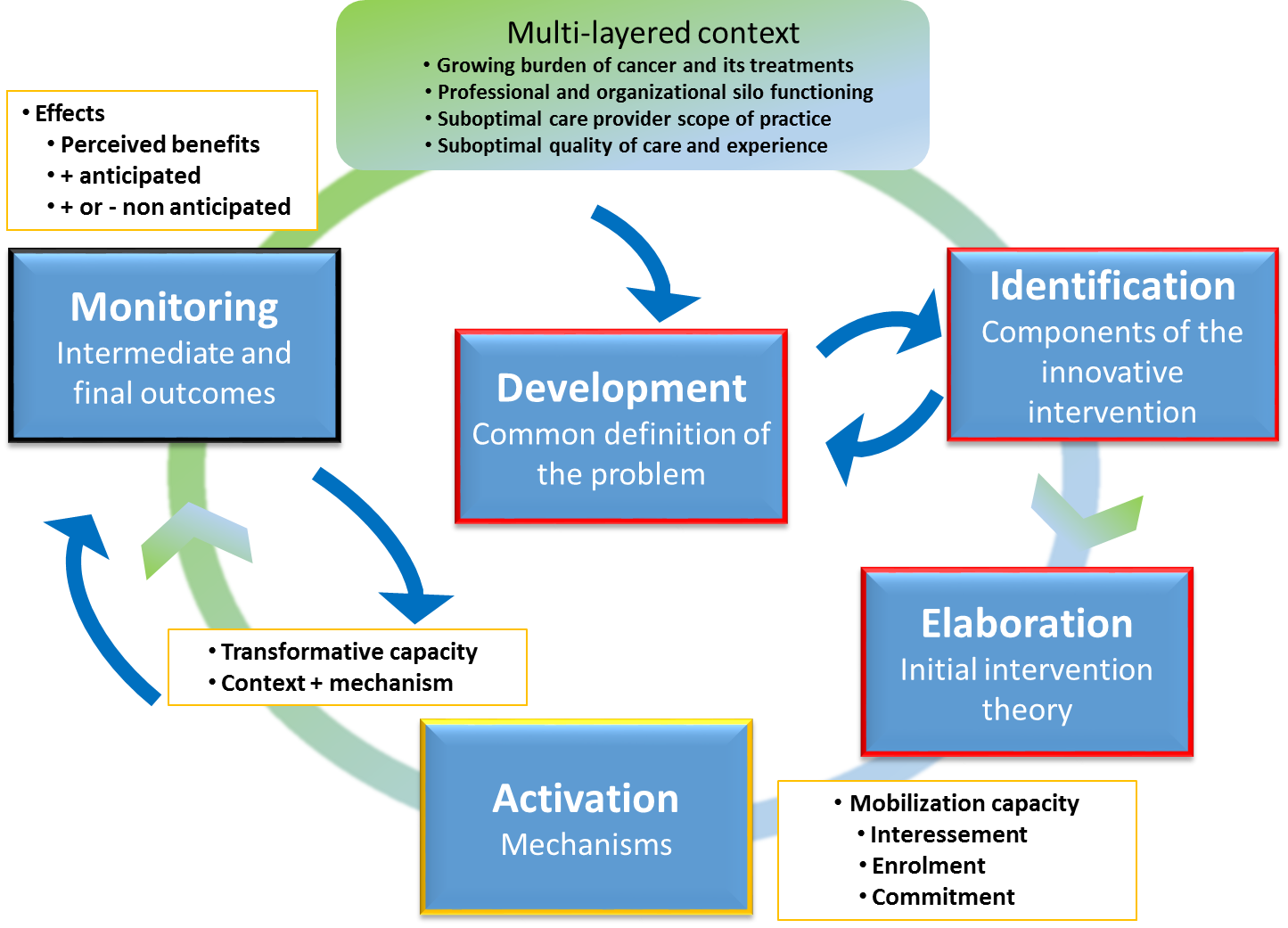


| **Elements** | **Definitions** |
| --- | --- |
| Multi-layered context | The context (**green box**) represents evolutionary dynamics of current practice. Contextualisation^1^, a stage in the translation of innovations into practice^1^, aims at identifying actors-actants (human and non-human), the stakes and dynamics that exert an influence at different levels of the health system (operational, managerial, governance). According to an *interventional research^2^* approach, researchers work closely with actors in the field to find concrete solutions to the problems they face. |
| Problem definition, intervention identification and elaboration of an initial theory | These actions (**red boxes**) refer to actors' perceptions of a problem and its solution as these take shape through the multiple components of the intervention. *Problematization*^1^ is a process of discussion and decision making that helps to clarify the nature and extent of the problem and determine the mechanisms to be activated in order to implement a solution. The result is an initial theory of the intervention which is fully aligned with the interventional research and integrated in the transfer of knowledge to practice^3^. |
| Activation of mechanisms | The *interessement*, *enrolment* and *commitment*^1^ of actors in their particular context lead to the activation of mechanisms (**yellow boxes**). This mobilization capacity fosters coordination of interdependent actors focusing on a shared goal. Activated mechanisms in context support transformative capacity. |
| **Monitoring** | Monitoring (**black box**) effects refers to the observation of intermediate (changes in professional practice) and final (patient experience of care) outcomes of the intervention. We are interested in actors’ perceptions of the benefits of the intervention by observing “*facts in the making*”. |
| **Configuration C + M = O** | The context-mechanisms-outcomes configuration can be developed according to the principles of Realist Evaluation^4^ to arrive at a refined theory of the intervention. |

**References**:

^1^ Callon M. Techno-economic networks and irreversibility. In: Law J, editor. A sociology of monsters: essays on power, technology, and domination. London: Routledge, 1991. pp 132-64.

^2^ Hawe P, Di Ruggiero E, Cohen E: Frequently asked questions about population health intervention research. Can J Public Health. 2012;103(6):e468-e71.

^3^ Canadian Institutes of Health Research (CIHR). Knowledge translation at CIHR. 2010. <http://www.cihr-irsc.gc.ca/e/29418.html>. Accessed 20 Sept 2015.

^4^ Pawson R, Tilley N. Realist evaluation. Magenta Text. 2004. <http://www.communitymatters.com.au/RE_chapter.pdf>. Accessed 20 sept 2015.
